# Decreased Trabecular Bone Score in Patients With Active Endogenous Cushing’s Syndrome

**DOI:** 10.3389/fendo.2020.593173

**Published:** 2021-01-21

**Authors:** Barbara Stachowska, Jowita Halupczok-Żyła, Justyna Kuliczkowska-Płaksej, Joanna Syrycka, Marek Bolanowski

**Affiliations:** Department and Clinic of Endocrinology, Diabetes and Isotope Therapy, Wroclaw Medical University, Wrocław, Poland

**Keywords:** trabecular bone score (TBS), bone mineral density (BMD), osteoporosis, fracture, FRAX, Cushing’s syndrome

## Abstract

**Introduction:**

The impairment in bone microarchitecture and reduced bone quality are relevant mechanisms underlying the increased fracture risk in Cushing’s syndrome (CS). The trabecular bone score (TBS) is a relatively novel textural index of bone microarchitecture.

**Purpose:**

The objective of the study was to compare TBS, bone mineral density (BMD), and fracture risk in patients with endogenous CS to controls. We have investigated the association of TBS with anthropometric parameters and 25(OH) vitamin D concentrations.

**Materials and Methods:**

The study group comprised 19 consecutive patients with CS (14 women and 5 men; mean age 45.84 ± 13.15 years) and sex-, age-matched 36 controls (25 women and men; mean age 52.47 ± 8.98 years). Anthropometric parameters, biochemical and hormonal data were compared between groups. Lumbar spine (L1–L4) and femoral neck BMD (LS BMD, FN BMD) measurements were performed. TBS values were obtained from lumbar spine DXA images.

**Results:**

TBS was significantly lower in patients with CS compared to controls (p = 0.0002). The 10-year probability of hip fracture and the 10-year probability of a major osteoporotic fracture were significantly higher in the CS group than in controls (p = 0.03, p < 0.0001, respectively). All subjects from the CS group with fractures had low TBS value (degraded microarchitecture). TBS correlated negatively with the duration of disease in patients with CS (r = -0.590 p = 0.008).

**Conclusions:**

The patients with active CS have altered bone microstructure as indicated by the decreased TBS and are at higher risk of hip and a major osteoporotic fractures. TBS seems to be a very important analytical tool facilitating fracture risk assessment in endogenous hypercortisolism.

## Introduction

Endogenous Cushing`s syndrome (CS) is characterized by oversecretion of glucocorticoids due to adrenocorticotropic hormone (ACTH)-secreting pituitary adenoma (in most cases of endogenous CS), ectopic ACTH production, or autonomous adrenal overproduction of cortisol (1). The incidence of endogenous CS is estimated at 0.2–5 per million population per year (2, 3). Excess cortisol secretion leads to a typical clinical presentation with all components of metabolic syndrome (diabetes mellitus, dyslipidemia, central obesity, and hypertension) as well as osteoporosis, muscle weakness, menstrual irregularities, and psychiatric dysfunction (4, 5). The occurrence of comorbidities including structural and functional alterations of the skeletal system is a relevant cause of increased mortality and impaired quality of life (6, 7). Osteoporosis is an underestimated and serious complication of endogenous CS with the prevalence ranging from 31.6% to approximately 50% among published studies (7–9). Patients with endogenous CS have an increased risk of fragility fractures, despite normal or slightly decreased bone mineral density (BMD). This could be explained by decreased bone strength due to the qualitative deterioration of bone structure (10–12). Dual-energy X-ray absorptiometry (DXA) is the most commonly used technique for the measurement of BMD and provides information concerning quantitative bone properties. However, DXA is inappropriate to estimate the quality of the bone tissue (13). The decreased reliability of BMD in predicting the fracture risk in CS may suggest that impairment in bone microarchitecture and reduced bone quality are relevant mechanisms underlying the increased fracture risk in subjects with hypercortisolism (14). Therefore, a non-invasive and widely available method to assess the microarchitecture of bone tissue is highly desired in patients with CS (15). The trabecular bone score (TBS) is a relatively novel textural index of bone microarchitecture derived from lumbar spine DXA images. It appears to be a parameter capable of providing indirect information on trabecular bone microarchitecture and bone quality (16, 17). TBS is associated with the trabecular bone volume/tissue volume (BV/TV), the number of trabeculae and their connectivity, and is negatively correlated with the space between trabeculae and with structure model index (18). A high TBS reflects a strong, fracture-resistant microarchitecture, whereas a low TBS represents a weak, fracture-prone microarchitecture (19). The added value of the TBS to BMD in fracture risk assessment was demonstrated in cross-sectional, prospective, and longitudinal studies (18). Recently, a large meta-analysis confirmed that TBS is a significant predictor of fracture risk independently of FRAX probability (20).

To date, the clinical relevance of TBS analysis was verified in several endocrinopathies associated with increased fracture risk, including disorders of the growth hormone/insulin-like growth factor 1 (GH/IGF-I) axis, diabetes type 1 and 2, primary hyperparathyroidism, and thyroid diseases (21). There are a few clinical studies documenting associations of autonomous cortisol secretion with TBS in endogenous CS of various etiologies (12, 22, 23). To the best of our knowledge, there is no study concerning TBS value analysis in endogenous CS in comparison to healthy controls.

The aim of this study was to compare TBS, BMD, and fracture risk in patients with endogenous CS to controls and investigate the association of TBS with anthropometric parameters and 25(OH) vitamin D concentrations.

## Materials and Methods

### Subjects

The study group comprised 19 consecutive patients with CS (14 women and 5 men; mean age 45.84 ± 13.15 years) and sex-, age-matched 36 controls (25 women and 11 men; mean age 52.47 ± 8.98 years). The participants (CS patients and controls) were recruited from the Department of Endocrinology, Diabetes and Isotope Therapy, Wroclaw Medical University. A thorough medical history was taken, including clinical fracture risk factors mentioned in the FRAX algorithm (exogenous glucocorticoids, alcohol intake, smoking habits, previous fracture, parent fractured hip, rheumatoid arthritis, secondary osteoporosis), drug history, and reproductive status. Patients’ weight (kg) and height (m) were recorded and their body mass index (BMI) was calculated. The following data were collected: results of basal and dynamic evaluation of the hypothalamic–pituitary–adrenal (HPA) axis, including ACTH concentrations, urinary free cortisol (UFC), cortisol circadian rhythm (serum cortisol measured at 6.00 a.m., 8.00 a.m., 8.00 p.m., at midnight), morning serum cortisol levels after the overnight 1 mg dexamethasone suppression test (1 mg DST) and suppression tests with 2 mg and 8 mg dexamethasone, 25(OH) vitamin D, dehydroepiandrosterone sulfate (DHEA-S), estradiol (E2), total testosterone (tT), calcium, and alkaline phosphatase (ALP). According to the Endocrine Society Clinical Guidelines, the CS diagnosis was established based on the following criteria: increased UFC (≥ 2 tests), serum cortisol levels greater than 1.8 μg/dl after 1 mg dexamethasone suppression test (DST), insufficient suppression of serum cortisol during low-dose dexamethasone suppression test (LDDST) (less than or equal to 1.8 μg/dl) (5). Patients with CS were divided into three groups on the basis of CS etiology: 14 patients with pituitary-dependent CS (P-CS), 2 patients with adrenal-dependent CS (A-CS) – CS from adrenal adenoma, 3 patients with CS from an ectopic source (one bronchial neuroendocrine tumor (atypical), one thymic carcinoid, one unknown source). No symptoms or signs of Cushing’s syndrome were presented in controls. Seven women with CS had hypogonadism, five women were postmenopausal. Women were considered hypogonadal when menstrual disturbances were noted and E2 concentrations were below the normal range for the early follicular phase. Two men had hypogonadism (tT concentrations below the normal reference range). Eight men in the control group had hypogonadism, eight women in the control group were postmenopausal. No patient had growth hormone deficiency or secondary hypothyroidism. Hyperprolactinemia was not observed in any participant. Diabetes mellitus type 2 treated with oral hypoglycemic medications was observed in eight CS patients and in two controls. Insulin therapy was required in four patients with CS. All participants had a normal renal and hepatic function. Patients with diagnosed adrenal cancer were excluded from the study. None of the subjects took drugs known to interfere with bone metabolism, medications for osteoporosis, oral contraceptives, exogenous glucocorticoids, or hormonal replacement therapy. The bioethics committee of Wroclaw Medical University approved the protocol of the study. All subjects signed informed consent forms in accordance with the Declaration of Helsinki.

### BMD Measurements

Lumbar spine (L1–L4) and femoral neck BMD measurements were performed using the DXA technique (Hologic densitometer). BMD measurements in all subjects (CS group and controls) were performed during their stay in our Department of Endocrinology, Diabetes and Isotope Therapy at the beginning of the study. The analyzed locations were at the lumbar spine (LS) and the femoral neck (FN). Results were presented as BMD (g/cm^2^), T-score and Z-score. In postmenopausal women and men aged >50 years, the following criteria for BMD loss were used: T-score ≥-1 standard deviations (SD) – normal; T-score between -1 and -2.5 SD – osteopenia; T-score ≤-2.5 SD – osteoporosis (24). In the patients with premenopausal status or younger than 50 years the value of BMD was considered as a Z-score: Z-score values of -2.0 SD or lower are stated “below the expected range for age” and those above -2.0 SD “within the expected range for age” (24).

### TBS Method

TBS values were obtained from lumbar spine DXA images using TBS iNsight software, version 3.0.3.0 (Med-Imaps, Pessac, France). According to the results from other studies, the TBS values were classified as follows: TBS ≥1.31 normal, 1.31–1.23 partially degraded microarchitecture, ≤1.23 degraded microarchitecture (25, 26).

### Assessment of the Fractures

To detect vertebral fractures, all patients with CS underwent lateral, thoracolumbar X-ray imaging. According to the Genant visual semiquantitative method, vertebral fractures were defined as reductions on lateral spine radiographs of 20% in one vertebral body’s height (27). An X-ray to detect vertebral fractures was performed in the CS group, but not in the controls. Data concerning previous fractures was obtained from medical history (traumatic fractures were detected through routine X-rays) for both the CS group and controls. Low-traumatic fractures were defined as those resulting from a fall from standing height or less (12). Traumatic fractures are result of trauma (i.e., fall from standing higher than height) or accident.

### Fracture Risk Estimation

The 10-year probability of hip fracture and the 10-year probability of a major osteoporotic fracture (hip, spine, wrist, or humerus) were assessed using on-line FRAX algorithm (http://www.shef.ac.uk/FRAX) (28, 29). We used the FRAX algorithm in patients with CS and control group and we included femoral neck BMD value to enhance fracture risk prediction (29). The FRAX algorithm is based on age, weight, height, sex, and clinical risk factors such as use of exogenous glucocorticoids, previous fracture, alcohol intake, smoking habits, family history of hip fracture, rheumatoid arthritis, or secondary osteoporosis [such as chronic malnutrition or malabsorption, type 1 diabetes, osteogenesis imperfecta, untreated long-standing hyperthyroidism, hypogonadism or premature menopause (< 45 years) and chronic liver disease] (29). The FRAX algorithm is designed for subjects aged 40–90 years, for younger subjects the program investigated probabilities for a patient aged 40 year (29).

### Laboratory Examinations

The E2, tT, DHEAS were measured by chemiluminescence immunoassay method using Immulite 2000 (Siemens Healthcare Diagnostics, USA). Reference ranges were as follows: E2 premenopausal women: follicular phase <160.0 pg/ml, ovulation phase 34–400.0 pg/ml, luteal phase 27.0–246.0 pg/ml; postmenopausal women <30.0 pg/ml; men <56.0 pg/ml (analytical sensitivity: 15.0 pg/ml); T premenopausal women 0.2–0.72 ng/ml, postmenopausal women 0.2–0.43 ng/ml, men 0.72–8.53 ng/ml, >50 years 1.29–7.67 ng/ml (analytical sensitivity: 0.15 ng/ml); DHEA-S: women 35.0–430.0 µg/dl; men 80.0–560.0 µg/dl (analytical sensitivity: 3.0 µg/dl). Cortisol and 25(OH)D concentrations were measured by chemiluminescent immunoassay using Architect i1000 (Abbott Laboratories, USA). Reference ranges: cortisol before 10 a.m. 3.7–19.4 μg/dl, after 5 p.m. 2.9–17.3 μg/dl. Limit of detection (LOD) was ≤0.8 µg/dl, analytical sensitivity was: 1.0 µg/dl. The ACTH was measured by chemiluminescence immunoassay method using Immulite 2000 (Siemens Healthcare Diagnostics, USA), reference ranges was: ACTH <46 pg/ml (analytical sensitivity: 5.0 pg/ml). The ranges of 25(OH)D concentrations were as follows: vitamin D deficiency <20 ng/ml, suboptimal status 20–30 ng/ml, optimal status 30–70 ng/ml. LOD was 2.2 ng/ml, limit of quantitation (LOQ) was 2.4 ng/ml. Urine free cortisol excretion was measured using a radioimmunoassay method (Immunotech, Beckman Coulter Inc., Prague, Czech Republic), reference range: 14.0–75.0 μg/24 h. Serum calcium and alkaline phosphatase were measured using colorimetric assays on an Architect c4000 (Abbott Laboratories, USA). Reference ranges were as follows: calcium 8.4–10.2 mg/dL (LOD: 0.5 mg/dl; LOQ: 1.0 mg/dl); alkaline phosphatase 40–150 IU/l (LOD: 5.0 IU/l; LOQ: 5.0 IU/l).

### Statistical Analysis

The statistical analysis was performed using R for Windows software (version 4.0; Foundation for Statistical Computing, Vienna, Austria). Variables were presented as mean with standard deviation (SD) and median with interquartile ranges (IQR). The Shapiro–Wilk’s test was used to determine the normality of the variables. Mann-Whitney test was applied to compare quantitative variables between study groups. Proportional differences were tested using Fisher’s exact test. Correlation between two quantitative variables was calculated using Spearman’s rank correlation test. Additionally, multiple regression analysis was performed to identify the predictors of TBS value. P-value <0.05 was considered significant.

## Results

**Figure 1:**
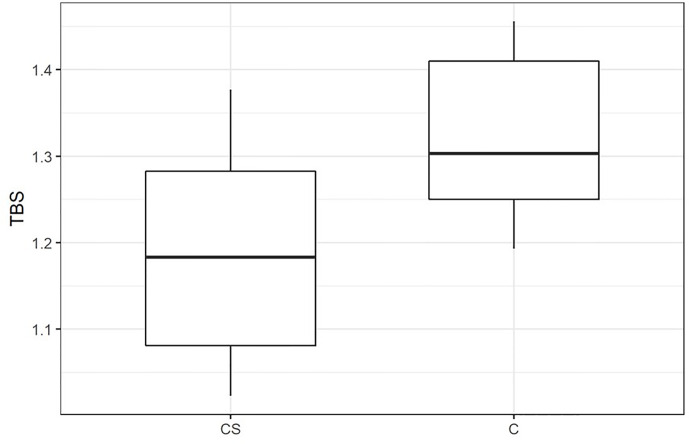
Trabecular bone score (TBS) in patients with CS and control group. CS, patients with Cushing’s syndrome; C, Controls; TBS, trabecular bone score, p < 0.001.

The general characteristics of the patients with CS and controls are presented in **Table 1**. There were no significant differences in age, sex, height, weight, and BMI between studied groups. ACTH, morning cortisol concentrations, and UFC were significantly higher in the CS patients than in controls (p < 0.0001). DHEA-S concentrations were also significantly higher in the CS group. 25(OH)D concentrations were significantly lower in the CS patients compared to controls (p = 0.03). LS and FN BMD measurements were significantly lower in patients with CS. TBS was significantly lower in patients with CS compared to controls (p = 0.0002) **Figure 1**. The 10-year probability of hip fracture and the 10-year probability of a major osteoporotic fracture were significantly higher in the CS group than in controls (p = 0.03, p < 0.0001, respectively). The median of the delay to CS diagnosis was 12 months (range 1–36 months) in the overall series of patients with CS. In the P-CS group, the median of the delay to diagnosis was 10 months (range 4–36), 12 months (range 1.5–24 months) in the A-CS, and 3 months in the E-CS. The median of time elapsed to diagnosis was significantly lower in the E-CS group compared with either the P-CS or the A-CS group (p < 0.01 for both comparisons).

**Table 1:** The general characteristics of the patients with Cushing’s syndrome and controls.

	CS (n = 19)			Controls (n = 36)			*p value*
	Mean ± SD	Median	IQR	Mean ± SD	Median	IQR	
Sex (female%/male%)	74/26%			70/30%			
Age (years)	45.8 ± 13.2	44.0	36.0–54.5	52.1 ± 9.0	51.5	42.8–61.3	0.06
High (cm)	167.3 ± 10.5	167	162.0–171.0	169.1 ± 8.3	167.5	163.8–175.2	0.6
Weight (kg)	77.7 ± 17.6	74.8	63–85.5	78.1 ± 15.6	77	64.8–88.5	0.7
BMI (kg/m²)	27.7 ± 4.8	28.0	23.5–29.7	27.2 ± 4	27.1	24.6–29	0.6
ACTH (pg/ml)							
Cortisol 08.00 (μg/dl)	26.4 ± 22.0	18.0	14.8–30.0	9.8 ± 2.8	9.6	7.9–11.2	<0.001
UFC (µg/24 h)	680.4 ± 699.9	503.00	232.8–819.0	51.1 ± 25.8	43.99	33.3–56.9	<0.001
Estradiol female (pg/ml)	41.3 ± 32.1	25.50	20.0–45.5	68.1 ± 96.2	32.35	23.3–51.5	0.2
Testosteron female (ng/ml)	1.2 ± 1.5	0.6	0.3–0.7	0.4 ± 0.6	0.2	0.2–0.3	<0.001
Testosteron male (ng/ml)	3.3 ± 1.7	3.2	2.3–4	3.2 ± 1.2	2.7	2.3–3.7	<0.001
DHEA-S (µg/dl)	315.9 ± 205.9	309	159.0–408.5	120.6 ± 77.5	113	61.2–156.5	<0.001
Serum calcium (mg/dl)	9.3 ± 0.46	9.15	9.0–9.5	9.3 ± 0.30	9.3	9.1–9.5	0.4
25OHD (ng/ml)	21.6 ± 8.32	19.00	15.25–27.00	25.7 ± 6.1	25.4	21.8–29.1	0.03
ALP (IU/l)	64.4 ± 17.41	65	54.00–69.0	60.2 ± 17.8	57	48.7–70.5	0.4
TBS	1.198 ± 0.1224	1.215	1.091–1.288	1.329 ± 0.0856	1.312	1.257–1.412	<0.001
LS BMD (g/cm²)	0.8 ± 0.2	0.8	0.8–0.9	1 ± 0.1	0.96	0.9–1.1	0.008
LS T-score	-1.6 ± 0.9	-1.7	-2.1–(-1.2)	-0.9 ± 1.0	-0.7	-1.6–0.1	0.02
LS Z-score	-1.1 ± 1.0	-1.0	-1.9–(-0.3)	0.1 ± 0.8	0.1	-0.7–0.7	<0.001
FN BMD (g/cm²)	0.7 ± 0.1	0.6	0.6–0.8	0.8 ± 0.1	0.8	0.7–0.9	0.03
FN T-score	-1.3 ± 1.1	-1.6	-1.9–(-0.9)	-0.4 ± 1.2	-0.6	-1.5–0.0	0.005
FN Z-score	-0.5 ± 1.2	-0.9	-1.2–(-0.1)	0.4 ± 1.1	0.3	-0.4–0.8	0.004
Hip Fracture FRAX (%)	1.4 ± 1.5	0.95	0.1–2.15	0.3 ± 0.3	0.2	0.2–0.5	0.03
Major osteoporotic Fractures FRAX (%)	6.9 ± 4.8	4.9	3.4–10.1	2.1 ± 0.9	1.8	1.5–2.7	<0.001
Low traumatic VF	10 (53%)			0			
Traumatic previous non-VF	3 (16%)			7 (19%)			

25(OH)D, 25(OH) vitamin D; BMD, bone mineral density; LS, lumbar spine; FN, femoral neck; UFC, urinary free cortisol; TBS, trabecular bone score; ALP, alkaline phosphatase; FRAX, Fracture risk assessment; BMI, body mass index; CS, Cushing’s syndrome; DHEA-S, dehydroepiandrosterone-sulfate; IQR, interquartile range; significant results (p < 0.05); Low traumatic VF, low traumatic vertebral (thoracic and lumbar vertebrae) fractures; Traumatic previous non-VF, traumatic previous non-vertebral fractures. p-value refers to the median.

### Comparison Between the Cushing’s Syndrome Patients With and Without Hypogonadism

There were no statistically significant differences in age, sex, BMI, 25(OH)D, ACTH, morning cortisol concentrations, and UFC between the CS patients with and without hypogonadism (p > 0.05). In the CS patients with hypogonadism significantly higher weight and height were noted (p = 0.007, p = 0.03, respectively). LS BMD, LS T-score, FN BMD, FN T-score, FN Z-score were significantly higher in patients with CS and concomitant hypogonadism (p = 0.03). TBS and fracture risk did not differ significantly between CS patients with and without hypogonadism.

## Comparison Between the Cushing’s Syndrome Patients With and Without Previous Fractures

Vertebral (thoracic and lumbar vertebrae) fractures were found in 10 (53%) patients (**Table 2**). Clinically symptomatic fractures were registered in 6 (60%) patients. They were associated with chronic back pain, decreased spinal mobility, and height reduction about 4–8 cm decreased of final stature. The location of fractures was mainly in thoracic and/or lumbar vertebrae. Rib and wrist fractures were detected in 3 patients. Nine patients with vertebral fractures had values of lumbar BMD within the normal range. All the patients with fractures had low TBS value (degraded microarchitecture) (**Table 2**).

**Table 2:** Bone status of CS subjects and every etiological group—prevalence of microarchitecture identified as degraded, partially degraded, or normal by lumbar trabecular bone score (TBS), low or high bone mineral density (BMD), and fractures.

	CS (n = 19)
TBS ≥ 1.31	3 (15.8%)
TBS ≤ 1.23	12 (63.2%)
TBS 1.23–1.31	4 (21.1%)
Low LS BMD	4 (21.1%)
Normal LS BMD	15 (78.9%)
Low TBS plus Low LS BMD	4 (21.1%)
Low TBS plus normal BMD	8 (42%)
Vertebral fractures plus low TBS	10 (52.6%)
Low traumatic vertebral fractures	10 (52.6%)
Non-vertebral low traumatic fractures	3 (15.8%)
Fractures plus low LS BMD	1 (5.3%)

TBS, trabecular bone score; CS, Cushing’s syndrome; BMD, bone mineral density; LS, lumbar spine; Fractures, vertebral (thoracic and lumbar vertebrae) fractures.

Low traumatic fractures were registered only in CS group. Traumatic non-vertebral previous fractures (1 kneecap, 2 phalanx, 1 radius, 2 nasal bone, 1 tibia) were registered in controls and in CS patients (1 radius, 1 nasal bone, 1 tibia).

There were no significant differences in age, sex, weight, BMI, and 25(OH)D between the CS patients with and without previous fractures. ACTH, morning cortisol concentrations, and UFC were higher in fractured patients but the difference did not reach statistical significance. No significant differences in BMD measurements were also observed. There was a trend to lower TBS values in patients with CS and a history of fractures (p = 0.06). The 10-year probability of hip fracture and the 10-year probability of major osteoporotic fracture were significantly greater in the CS patients with a history of fractures (p = 0.0009, p = 0.002, respectively).

### Trabecular Bone Score Associations With Hormones, Demographic, and Anthropometric Factors

The Bivariate analysis showed that TBS was inversely correlated with age in patients with CS and in controls (r = -0.691 p = 0.001; r = -0.601 p = 0.0001, respectively). TBS correlated negatively with the duration of disease in patients with CS (r = -0.590 p = 0.008). A negative correlation between TBS and BMI was observed in the CS group (r = -0.610 p = 0.006). TBS correlated positively with height and DHEA-S in controls (r = 0.350 p = 0.037; r = 0.640 p < 0.0001, respectively). There were no significant correlations between TBS and weight, E2, T, 25(OH)D, calcium, ALP, ACTH, and morning cortisol concentrations in both groups, CS and controls (**Table 3**).

**Table 3:** TBS associations with hormones, demographic, and anthropometric factors.

	CS	Controls
	*p value*	*p value*
Age (years)	0.001	0.0001
Weight (kg)	0.1977	0.037
High (cm)	0.4793	0.0365
BMI (kg/m^2^)	0.006	0.5992
ACTH (pg/ml)	0.9233	0.8958
Cortisol 08.00 (μg/dl)	0.3330	0.1802
UFC (μg/24 h)	0.0405	0.8345
Estradiol female (pg/ml)	0.6682	0.1921
Testosteron female (ng/ml)	0.4643	0.2973
Testosteron male (ng/ml)	0.1041	0.4160
DHEA-S (μg/dl)	0.6497	0.0001
Serum calcium (mg/dl)	0.7268	0.7606
25OHD (ng/ml)	0.7642	0.0986
ALP (U/l)	0.6888	0.3079
BMD Lumbar Total (g/cm^2^)	0.4503	0.001
T-score Lumbar Total	0.8581	0.001
Z-score Lumbar Total	0.2943	0.0205
BMD neck (g/cm^2^)	0.8250	0.0907
T-score Neck	0.7309	0.0303
Z-score Neck	0.8751	0.4202
Hip Fracture - FRAX (%)	0.1543	0.0032
Major osteoporotic - FRAX (%)	0.0406	0.0028
Duration of the disease	0.008	

25(OH)D, 25(OH) vitamin D; BMD, bone mineral density; LS, lumbar spine; FN, femoral neck; UFC, urinary free cortisol; TBS, trabecular bone score; ALP, alkaline phosphatase; FRAX, Fracture risk assessment; BMI, body mass index; CS, Cushing’s syndrome; DHEA-S, dehydroepiandrosterone-sulfate; significant results (p < 0.05), p-value refers to the median.

To identify baseline parameters that predict TBS value stepwise multivariate correlation analyses was performed. TBS significantly correlates with UFC, morning cortisol concentrations (p = 0.02, β = -0.4189; p = <0.001, β = -0.503743, respectively) and DHEAS level (p = 0.04, β = 0.4187) in both groups. A positive correlation between TBS and LS BMD was noticed in controls (r = 0.713 p < 0.0001). There were no statistically significant correlations between TBS and LS BMD in the group of patients with CS and between TBS and FN BMD in both group (**Table 4**).

**Table 4:** Multiple linear regression analyses of the associations of UFC, morning cortisol levels, DHEA-S with TBS.

	Estimate	β	SE	*p value*
UFC (µg/24 h)	-0.0002133	-0.4189732	0.0000865	0.02
Cortisol 8.00 (µg/dl)	-0.009574	-0.503743	0.002487	<0.001
DHEA-S (µg/dl)	0.00472	0.418708	0.0000969	0.044

β, standardized regression coefficient; Estimate, unstandardized regression coefficient; SE, standard error; β, standardized regression coefficient; UFC, free urine cortisol; DHEA-S, dehydroepiandrosterone-sulfate; TBS, trabecular bone score, significant results (p < 0.05).

### Bone Mineral Density Associations With Hormones, Demographic, and Anthropometric Factors

The Bivariate analysis showed that Lumbar BMD was inversely correlated with age in controls but not in CS group (r = -0.5264, p = 0.0010). BMD Lumbar correlated positively with weight (r = 0.5569, p = 0.0133, r = 0.4110, p = 0.0128, respectively) and height (r = 0.5051, p = 0.0274; r = 0.4762, p = 0.0033 respectively) in CS and controls. BMD correlated positively with E2 and DHEA-S in controls (r = 0.5427, p = 0.0051; r = 0.5774, p = 0.0002, respectively). There were no significant correlations between BMD Lumbar and T, 25(OH)D, calcium, ALP, ACTH, UFC, and morning cortisol concentrations in both groups, CS and controls.

### FRAX Associations With Trabecular Bone Score and Bone Mineral Density

The 10-year probability of a major osteoporotic fracture was inversely correlated with TBS in CS and controls (r = -0.5162, p = 0.0406; r = -0.4836, p = 0.0028, respectively). There were no significant correlations between the 10-year probability of a major osteoporotic fracture and LS BMD in CS group. The 10-year probability of a major osteoporotic fracture was inversely correlated with BMD Lumbar in controls (r = -0.5691, p = 0.0003). The 10-year probability of a major osteoporotic fracture correlated positively with age (r = 0.8087, p = 0.001, r = 0.6573, p = 0.0057) and inversely with height (r = -0.5398, p = 0.0309; r = -0.5915, p = 0.0001, respectively) and weight (r = -0.551, p = 0.0270, r = -0.602, p = 0.0001, respectively) in CS and controls. There were no significant correlations between the 10-year probability of a major osteoporotic fracture and 25(OH)D, calcium, ALP, ACTH, UFC and morning cortisol concentrations in both groups, CS and controls.

## Discussion

This is the first study comparing TBS values in patients with active CS and healthy subjects without signs and symptoms of CS. Obtained results indicate clearly that CS patients had lower TBS. Previously, Belaya et al. demonstrated that the mean value of TBS was 1.207 in a cohort of patients with active endogenous CS and is lower than in the general healthy population (12). A study on 102 patients with adrenal incidentalomas (AIs) showed that subjects with subclinical hypercortisolism (SH) had lower TBS than patients without SH and controls (22). Accordingly, Vinolas et al. reported lower TBS but similar BMD in patients with mild autonomous cortisol secretion (MACE) than in patients with nonsecreting adrenal incidentalomas (NSA). Degraded or partially degraded microarchitecture indicated by TBS were observed in 52% of MACE patients and in 33% of patients with NSA (23). A reduced TBS was also noted in glucocorticoid treated women and men (13, 30). The authors exhibited that TBS alone and LS BMD+TBS, but not LS BMD alone, were capable of discriminating between glucocorticoid-treated and glucocorticoid-naive women (13).

Our study showed that TBS was significantly lower in CS patients with fractures and was correlated with the 10-year probability of a major osteoporotic fractures. BMD was not associated with the risk of major osteoporotic fractures. Vertebral fractures were found in 53% of CS patients. The majority of patients (90%) with vertebral fractures had values of LS BMD within the normal range but all the patients with fractures had low TBS value (degraded microarchitecture). These findings are a confirmation of altered microarchitecture in overt cortisol excess conditions. Vinolas et al. revealed that fragility fractures tended to be associated with low TBS but not BMD (23).

We did not find a significant difference in TBS values between hypogonadal and eugonadal patients with CS as well as between fractured CS patients and patients without fracture. However, there was a trend to lower TBS in fractured subjects. Belaya et al. reported no significant differences in mean TBS between patients with and without fragility fractures (12). A trend towards an increased frequency of degraded and partially degraded microarchitecture (altered TBS) in patients with fragility fractures compared to patients without fractures was reported by Vinolas et al. Moreover, these authors did not observe a significant difference in TBS and BMD between hypogonadal and eugonadal patients with overt CS and MACE. They suggest that the negative effect of hypercortisolism overwhelms the bone-protecting effects of sexual hormones (23). We noted an inverse association between TBS value and patients’ age, which is in line with previously published studies (16, 17, 31, 32). A significant decrease of TBS was noted with ageing in healthy Caucasians. BMI did not influence TBS in both male and female (31). In our study inverse association between TBS and BMI was presented in the CS group. McCloskey et al. created a meta-analysis of studies on men and women with different ethnic origins and demonstrated an overall weaker negative correlation between TBS and BMI (20). In contrast, BMI has been shown to be positively associated with lumbar spine (trabecular) volumetric BMD (LS-VBMD) on QCT (33). These findings suggest a more complex association between BMI, TBS, and BMD. Langsetmo et al. reported the limited clinical utility of TBS among older men with high BMI or high trunk lean mass (34).

Van Staa et al. in a meta-analysis of the effects of glucocorticoid therapy on bone showed that cumulative rather than daily doses of glucocorticoids correlated with BMD values (35). The cumulative dose is determined by the duration of the therapy and daily dose (35). Endogenous hypercortisolism may influence bone by both its severity and duration (35). The duration of CS prior to its diagnosis plays an important role in the intensity of glucocorticoid-related bone alterations. Our data suggests a strong association between TBS value and time elapsed to diagnosis. CS has to be diagnosed and treated as early as possible. Proper diagnosis is important to reduce complications of comorbidities like vertebral fractures.

In our study patients with CS had lower BMD compared to controls, which could be an obvious result of cortisol excess. Decreased BMD is a well-known factor contributing to an increase in fracture risk (36). However, it should be mentioned that normal or only slightly decreased BMD were also reported in other studies (12, 37, 38). That is why the evaluation of TBS additionally to BMD may be of great relevance in a clinical practice. Belaya et al. reported decreased LS and FN BMD in CS patients with fractures compared with patients without fracture (12). We observed a similar trend, however, the result did not achieve statistical significance. No difference in BMD was found between patients with and without fractures in other study (23). In our study, only 21.1% of the CS patients displayed low BMD. This data is consistent with the result from the study of Vinolas et al. (23). Importantly, all CS patients with decreased BMD also had decreased TBS. Moreover, 42% of the CS patients with normal BMD exhibited decreased TBS. Clinical presentation of bone status in CS patients revealed that endogenous hypercortisolism leads to low TBS value but to only a minimal decrease in BMD (12).

In the current study, we investigated fracture risk in patients with CS using the FRAX algorithm. The 10-year probability of hip fracture and the 10-year probability of a major osteoporotic fracture were higher in patients with CS compared to controls. Furthermore, there was no significant difference in fracture risk between CS patients with and without hypogonadism. Previously, Trementino et al. asked the question: is the FRAX algorithm useful in endogenous hypercortisolism? The authors suggested applying the FRAX algorithm to all active CS patients to better identify patients at higher risk of fracture. Moreover, they indicated that “fracture threshold” of 17% for the 10-year probability of major osteoporotic fracture might represent the “intervention threshold” in patients with CS (39). Still, there is no data concerning the validation of the FRAX method in CS patients (40). The studies analyzing the utility of the FRAX algorithm in endogenous hypercortisolism are highly needed.

Vitamin D deficiency is a common health problem (41). We observed decreased 25(OH) vitamin D concentrations in patients with CS. This result is in accordance with a previous study conducted on 37 cases (age > 18 years) of endogenous hypercortisolemia and 48 healthy controls (36). In a study on patients with adrenal incidentalomas, no significant differences in 25(OH) vitamin D between patients with and without subclinical hypercortisolism were demonstrated. According to our findings 25(OH)D level was comparable between fractured and non-fractured patients with subclinical hypercortisolism (22). We did not reveal a significant correlation between TBS and 25(OH) vitamin D concentrations. Similarly, other authors reported that 25(OH)D level was not associated with TBS and cortisol excess (22). Boyd et al. documented a weak association between 25(OH)D and bone microarchitecture measured by high-resolution peripheral quantitative computed tomography (HRpQCT) in a population of mostly vitamin-D-sufficient participants (42).

Like in other studies on CS, the main limitation of our study was a small number of patients with CS which can be explained by the rarity of endogenous cortisol excess. The second limitation of our study is the fact that data was obtained from patients at a single medical center. Future prospective multicenter studies of independent cohorts should include more individuals with CS.

In conclusion, the patients with active CS have altered bone microstructure as indicated by the decreased TBS and are at higher risk of hip and a major osteoporotic fractures. TBS seems to be a very important analytical tool facilitating fracture risk assessment in endogenous hypercortisolism.

## Data Availability Statement

The original contributions presented in the study are included in the article/supplementary materials; further inquiries can be directed to the corresponding author.

## Ethics Statement

The Bioethics Committee of Wroclaw Medical University approved the protocol of the study. All subjects signed informed consent forms in accordance with the Declaration of Helsinki. The patients/participants provided their written informed consent to participate in this study.

## Author Contributions

All authors contributed to the study conception and design. Material preparation, data collection, and analysis were performed by BS and JH-Z. The first draft of the manuscript was written by BS, and all authors commented on previous versions of the manuscript. BS conceptualized the study. JH-Z contributed to the methodology. JH-Z, JK-P and MB performed the Formal analysis and investigation. JS performed the BMD and TBS analysis. BS and JH-Z wrote and prepared the original draft. JK-P and MB wrote, reviewed, and edited the manuscript. All authors contributed to the article and approved the submitted version.

## Funding

This study was supported by grant number SUB.C120.20.016 (Minister of Science and Higher Education).

## Conflict of Interest

The authors declare that the research was conducted in the absence of any commercial or financial relationships that could be construed as a potential conflict of interest.
